# Association of CXCL13 and Immune Cell Infiltration Signature in Clear Cell Renal Cell Carcinoma

**DOI:** 10.7150/ijms.46874

**Published:** 2020-06-27

**Authors:** Fangdong Jiao, Hao Sun, Qingya Yang, Hui Sun, Zehua Wang, Ming Liu, Jun Chen

**Affiliations:** Department of Urology, Qilu Hospital (Qingdao), Cheeloo College of Medicine, Shandong University, 758 Hefei Road, Qingdao, Shandong, 266035, China.

**Keywords:** Clear cell renal cell carcinoma, immune cell infiltration signature, CXCL13, TCGA

## Abstract

Clear cell renal cell carcinoma (ccRCC) is one of the most commonly diagnosed kidney tumors and is often accompanied by immune cell infiltration. In this study, we attempted to identify microenvironment-associated genes and explore the correlation between CXCL13 and tumor-infiltrating immune cells (TIICs). Gene expression profiles and their corresponding clinical information were downloaded from The Cancer Genome Atlas (TCGA) database. The ESTIMATE (Estimation of Stromal and Immune cells in Malignant Tumor tissues using Expression data) algorithm was used to calculate immune cell and stromal cell scores, according to which patients were divided into high- and low-score groups, allowing differentially expressed genes (DEGs) to be identified. Functional enrichment and PPI network analysis were used to identify the functions of the DEGs. CIBERSORT algorithm and TIMER analysis were used to evaluate the immune score. Oncomine and TCGA database were used to explore CXCL13 mRNA expression level in ccRCC. High ESTIMATE score was significantly associated with prognosis. Functional enrichment analysis clarified that DEGs were associated with T cell activation, immune response-regulating cell surface receptor signaling pathway, and positive regulation of cytokine production. PPI network was used to identify CXCL13 as a hub gene. And CIBERSORT algorithm and TIMER analysis showed that strong correlation between CXCL13 expression level and TIICs. Oncomine database was used to validate high CXCL13 expression level in ccRCC tissue, compared to normal tissues. In conclusion, we obtained a list of tumor microenvironment-related genes and identified CXCL13 as an immune response biomarker in patients with ccRCC, GSEA analysis, wound healing and transwell assays showed CXCL13 played a role in tumor migration.

## Introduction

Renal cell carcinoma (RCC) is the one of the most common types of kidney tumor, and remains a major global medical problem despite the numerous new treatment options available 1. Among these RCCs, the most common subtype is clear cell RCC (ccRCC) 2. Despite the novel, targeted immune drug therapy has increased the prognosis of patients, elucidating the mechanism of RCC remains vital. Blocking the interaction between programmed death-1 (PD-1)/programmed death-ligand 1 (PD-L1) has been shown to improve the prognosis of RCC 3, whilst promoting T-cell proliferation and survival by using high-doses of interleukin-2 has an objective response in approximately 10-25 % of patients 4. These results reflect the sensitivity of RCC to immunotherapy. RCC is often accompanied by the tumor-infiltrating immune cells (TIICs), including macrophages and lymphocytes 5. Infiltration by lymphocytes is an immune reaction associated with the elimination of cancer cells and thus improved prognosis in most cancers, including lung cancer, colorectal cancer, and ovarian cancer 6. However, higher levels of tumor-infiltrating lymphocytes usually indicates a poor prognosis and reduced survival in RCC 7, 8. These findings suggest that the tumor microenvironment plays a crucial role in the occurrence and development of RCC.

The tumor microenvironment consists of immune cells, mesenchymal cells, endothelial cells, extracellular matrix (ECM) molecules, and inflammatory mediators 9. Studies have shown that the tumor microenvironment affects the gene expression of tumor tissues and patient outcome, and therefore has a diagnostic and prognostic value for tumors 10-13. Immune and stromal cells make up the non-tumor components of the tumor microenvironment. The ESTIMATE (Estimation of Stromal and Immune cells in Malignant Tumor tissues using Expression data) algorithm has been developed to predict tumor purity using gene expression data 12. By analyzing the specific gene expression signature of a tumor, ESTIMATE can calculate immune cell and stromal cell scores to predict non-tumor cell infiltration. Other specific gene expression signature-based algorithms have been shown to be effective when applied to colon cancer 14, breast cancer 15, prostate cancer 16 and glioblastoma 13. Furthermore, a newly computational algorithm, CIBERSORT, was used to enumerate 22 immune cell subsets based on 547 key genes, and could reveal the several TIICs expression levels in different groups.

Several prognostic factors are currently available for RCC, including tumor staging, lymph node involvement, histological subtypes, and Fuhrman grading which are gold standard to predict the prognosis of RCC. Despite these biomarkers were used for early detection or prognosis, we still needed another biomarkers to enlarge the prognosis network of RCC 17. The Cancer Genome Atlas (TCGA) has been established to map the genome variations of human cancers using genomic analysis techniques, providing a wealth of clinical and expression profile data 18, 19. In this study, we used the dataset of patients with ccRCC from the TCGA database, ESTIMATE algorithm to evaluate the effect of non-tumor cell infiltration on prognosis, identifying a series of microenvironment-related genes, and CIBERSORT algorithm to quantify the proportions of immune cells.

In this study, we identified CXCL13 is associated with TIICs and prognosis of ccRCC patients. And GSEA analysis showed CXCL13 might play an important role in tumor migration.

## Materials and Methods

### Database and identification of microenvironment-associated genes

The gene expression profiles and clinical data of patients with ccRCC was obtained from the TCGA data portal (https://tcga-data.nci.nih.gov/tcga/) and the ESTIMATE algorithm was used to calculate immune cell and stromal cell scores from the downloaded data. Data analysis was performed using the R software package, limma 20. A fold change of > 1.5 and false discovery rate (FDR) of < 0.05 were used as cutoffs to identify differentially expressed genes (DEGs). Volcano plots and heat maps were generated using the ggplot2 and pheatmap packages, respectively, whilst the Venn diagram package was used to identify overlapping genes.

### DEG enrichment analysis and protein-protein interaction (PPI) network construction

Gene ontology (GO) enrichment analysis and Kyoto Encyclopedia of Genes and Genomes (KEGG) pathway enrichment analysis were performed using DAVID (The Database for Annotation, Visualization and Integrated Discovery) 21. An FDR of < 0.05 was used as the cut-off value. The STRING database 22 and cytoscape software 23 were used to retrieve and reconstruct a PPI network. Important nodes and subnetworks were predicted and explored using cytohubba, a cytoscape plugin 24, and the top 10 hub genes were selected from the results of each method.

### Assessment of immune cell type fractions

To quantify the proportions of immune cells in the ccRCC samples, the analytical tool called CIBERSORT and the LM22 gene signature were used in this study (https://cibersortx.stanford.edu/). The LM22 gene signature contains 547 genes that distinguish 22 human hematopoietic cell phenotypes, with the analysis conducted with 1,000 permutations 25. These TIICs included B cells (memory and naïve B cells), dendritic cells(activated and resting dendritic cells), macrophages (M0, M1 and M2 macrophages), 7 T-cell types (T follicular helper cells, resting memory CD4^+^ T cells, activated memory CD4^+^ T cells, naïve CD4^+^ T cells, gamma delta T cells, CD8^+^ T cells and T regulatory cells), natural killer cells (resting natural killer and activated NK cells), mast cells (resting and activated mast cells), monocytes, plasma cells, neutrophils and eosinophils. The CIBERSORT values generated were defined as immune cell infiltration fraction per sample. For each sample, the sum of 22 evaluated immune cell type fractions equaled 1.

### TIMER Database analysis

TIMER is a comprehensive resource for systematic analysis of immune infiltrates across diverse cancer types (https://cistrome.shinyapps.io/timer/) 26. It applies a deconvolution previously published statistical method to infer the abundance of TIICs from TCGA cohort, including 10,897 samples across 32 cancer types 27.

### Oncomine Database analysis

The expression level of CXCL13 in ccRCC was identified in the Oncomine database (https://www.oncomine.org/resource/login.html) 28. The threshold was determined according to the following values: p value of 0.05, fold change of 2.0.

### Gene set enrichment analysis (GSEA) analysis

GSEA was used to further understand CXCL13-related pathways. The expression level of CXCL13 was used as the phenotype annotation and ccRCC patients in the TCGA cohort were dichotomized into low and high categories. GSEA version 3.0 software (www.broadinstitute.org/gsea) was used to analyze data. The Molecular Signatures Database (MSigDB) of c5 (c5.all.v6.2.symbols.gmt) was used to assess the functional differences 29, 30. FDR < 0.01 was used as the cut-off criteria.

### Cell culture and reagents

All cells were obtained from ATCC. 786-O and OS-RC-2 cells were cultured in RPMI-1640 medium (Gibco) containing 10% fetal bovine serum (Gibco). Recombinant human CXCL13 was purchased from PeproTech.

### Wound healing and transwell assay

Cell migration was determined by wound healing assay. Briefly, CXCL13-treated and control cells in 6-well plates were wounded by using sterile 200 μl pipette tip for 20h. The speed of wound closure was determined by Photograph. Meanwhile, polycarbonate membrane filters of the chamber were used for the transwell assay. 200 μl cells suspension at a density of 5×10^5^ cells/ml in serum-free medium were seeded into the upper part of the chamber. And the lower chamber was filled with 600 μl medium containing 10% fetal bovine serum. After 24 h of incubation at 37 °C, and the migrated cells adhered to the bottom of the upper chamber were fixed in methanol and stained with crystal violet, photographed and counted via Image J.

### Statistical analysis

The Statistical Package for Social Science (SPSS version 23.0) program was performed in this study. To investigate potential risk factors for overall mortality, a log-rank test was performed during Kaplan-Meier survival analysis. Correlation analysis was assessed by using chi-square test and Pearson's correlation coefficient test. The two-tailed p value was used in this study, and a p value of <0.05 considered statistically significant (* p<0.05, ** p<0.01, *** p<0.001 and **** p<0.0001).

## Results

### Immune cell and stromal cell scores are associated with renal cell carcinoma survival

**Figure 1:**
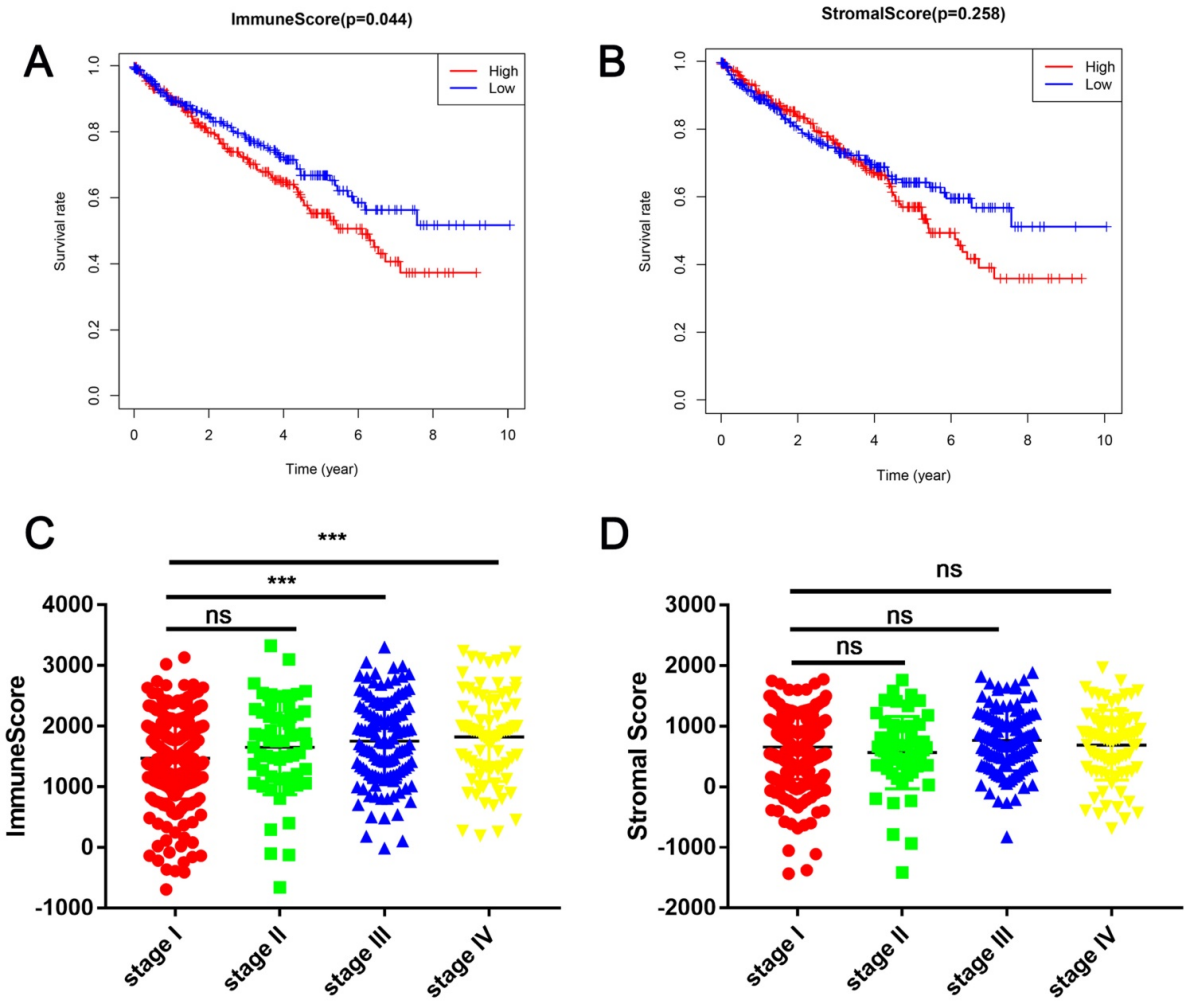
Immune cell and stromal cell scores are associated with renal cell carcinoma (RCC) survival (**A**) Patients with RCC were divided into two groups based on their immune cell scores. As shown in the Kaplan-Meier survival curve, patients with low immune cell scores had a higher overall survival than those with high immune cell scores (hazard ratio [HR] 1.372; 95 % CI 1.01-1.864; *P* = 0.0430 by log-rank test). (**B**) In a similar manner, patients with RCC were divided into two groups based on their stromal cell scores. The Kaplan-Meier survival curve shows no statistically significant difference between the two groups (*P* = 0.2547 by log-rank test). (**C**) Immune cell scores were higher in patients with a higher pathological stage. (^***^P < 0.001, by one-way ANOVA followed by Tukey's multiple-comparison post-hoc test). (**D**) The stromal cell scores showed no statistically significant differences at different pathological stages.

In this study, the clinical information and gene expression profiles of 530 patients with RCC were downloaded from the TCGA database. Of these patients, 188 (35.5 %) were females and 342 (64.5 %) were males, while 265 (50 %) cases were stage I, 55 (10.3 %) were stage II, 124 (23.4 %) were stage III, 83 (15.7 %) were stage IV, and 3 (0.6 %) were of unknown stage. The ESTIMATE algorithm was used to calculate immune cell and stromal cell scores to predict the infiltration of non-tumor cells. Next, we divided the patients with RCC into high- and low-score groups based on the median immune cell and stromal cell scores. The clinicopathologic characteristics of the 530 patients are shown in Table 1. Kaplan-Meier survival analysis revealed that patients with low immune cell scores had a higher survival rate than those with high immune cell scores (Figure 1A). Similarly, patients with lower stromal cell scores had increased overall survival (Figure 1B), although there was no statistically significant difference. Next, we analyzed the relationship between these scores and clinical stage, revealing that immune cell scores were higher in patients with a higher pathological stage (Figure 1C).

### Comparison of the gene expression profiles of patients with RCC with different immune cell and stromal cell scores

**Figure 2:**
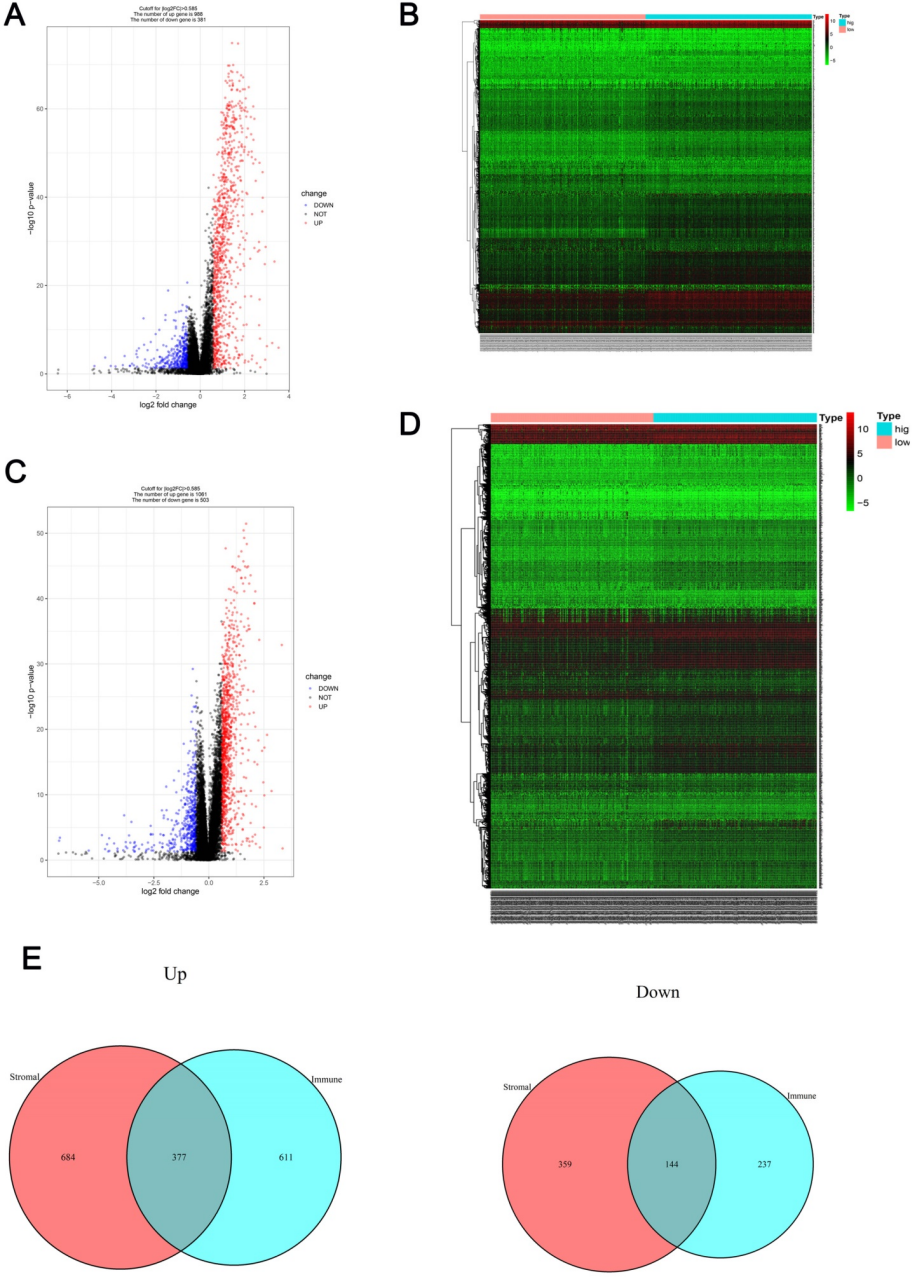
Gene expression profile comparison of patients with RCC with different immune cell and stromal cell scores (**A-B**) The volcano plot and heatmap show the 1369 genes (988 up-regulated and 381 down-regulated) identified based on the immune cell scores. (**C-D**) The volcano plot and heatmap show the 1564 genes (1061 up-regulated and 503 down-regulated) identified based on the stromal cell scores. (**E**) Venn diagrams show the 377 co-upregulated and 144 co-downregulated genes.

Patients were divided into low- and high-immune cell score groups and their gene expression profiles were analyzed to identify DEGs with corrected P-values < 0.05 and FDRs > 1.5. A total of 1369 DEGs (988 up-regulated and 381 down-regulated) were identified (Figure 2A) and visualized using a heatmap (Figure 2B). Using a similar method with the stromal cell scores, 1564 DEGs (1061 up-regulated and 503 down-regulated) were identified (Figure 2C, D). The co-regulated DEGs (377 co-upregulated and 144 co-downregulated) were visualized using Venn diagrams (Figure 2E).

### GO and KEGG pathway enrichment analysis of DEGs

**Figure 3:**
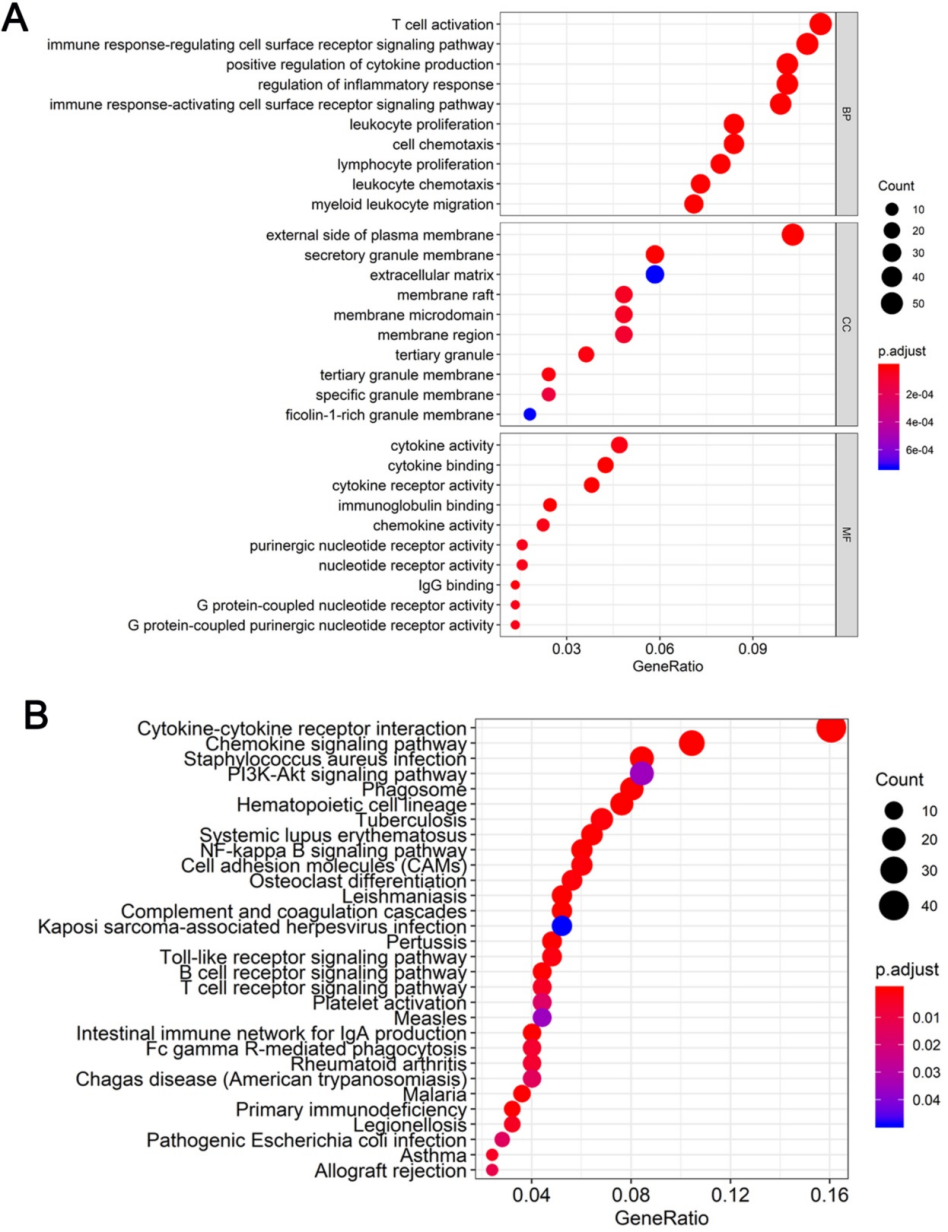
Functional enrichment analysis of DEGs (**A**) Top 10 biological process, cellular component, and molecular function terms for the co-DEGs. (**B**) KEGG pathways enriched for the co-DEGs.

To determine the functions of the 521 DEGs, we performed GO enrichment analysis; the top 10 GO terms are shown in Figure 3A. DEGs were enriched for T cell activation, immune response-regulating cell surface receptor signaling pathway, and positive regulation of cytokine production in the biological process category, the external side of the plasma membrane, secretory granule membrane, and extracellular matrix in the cellular component category, and cytokine activity, cytokine binding, and cytokine receptor activity in the molecular function category. We also performed KEGG pathway enrichment analysis to determine the pathways most enriched for DEGs, which included cytokine-cytokine receptor interactions, chemokine signaling pathways, and the PI3K-AKT signaling pathway (Figure 3B).

### PPI network analysis and identification of prognosis-associated genes

**Figure 4:**
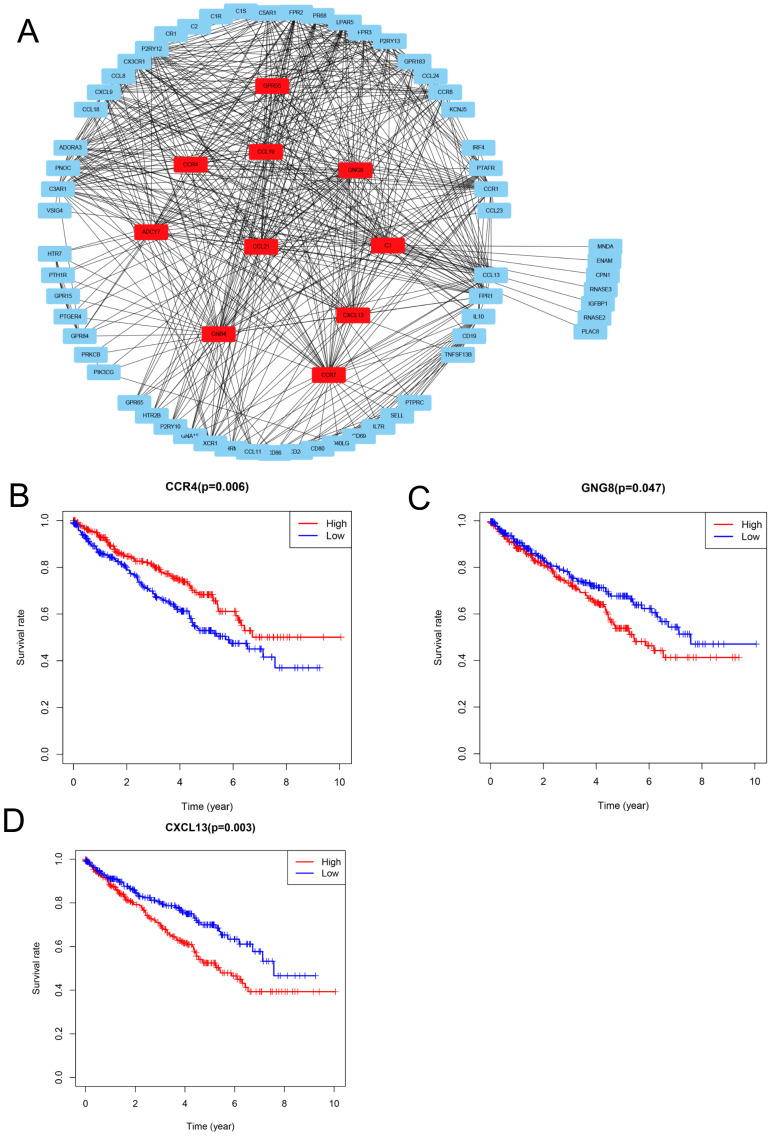
PPI network analysis and identification of prognosis-associated genes (**A**) Protein-protein interaction networks of the co-DEGs. (**B-D**) Kaplan-Meier plotters and log-rank tests for the prognostic value of DEGs. (B) CC4, (C) GNG8, (D) CXCL13.

To explore the relationships between the DEGs, The Search Tool for the Retrieval of Interacting Genes (STRING) database and Cytoscape software were used to construct a PPI network for the DEGs (Figure 4A). The important nodes and subnetworks of the PPI were predicted and explored using CytoHubba; the 10 most significant node genes were ADCY7, GPR55, CCR4, GNG8, GNB4, C3, CCL21, CCR7, CCL19, and CXCL13.

Since high levels of immune cell infiltration can reduce RCC prognosis, we identified genes affecting the prognosis of patients with RCC among top 10 hub genes. As shown in Figure 4B-D, CCR4, GNG8 and CXCL13 were associated with prognosis of ccRCC patients.

### The distribution of TIICs in ccRCC according to CXCL13 expression level

**Figure 5:**
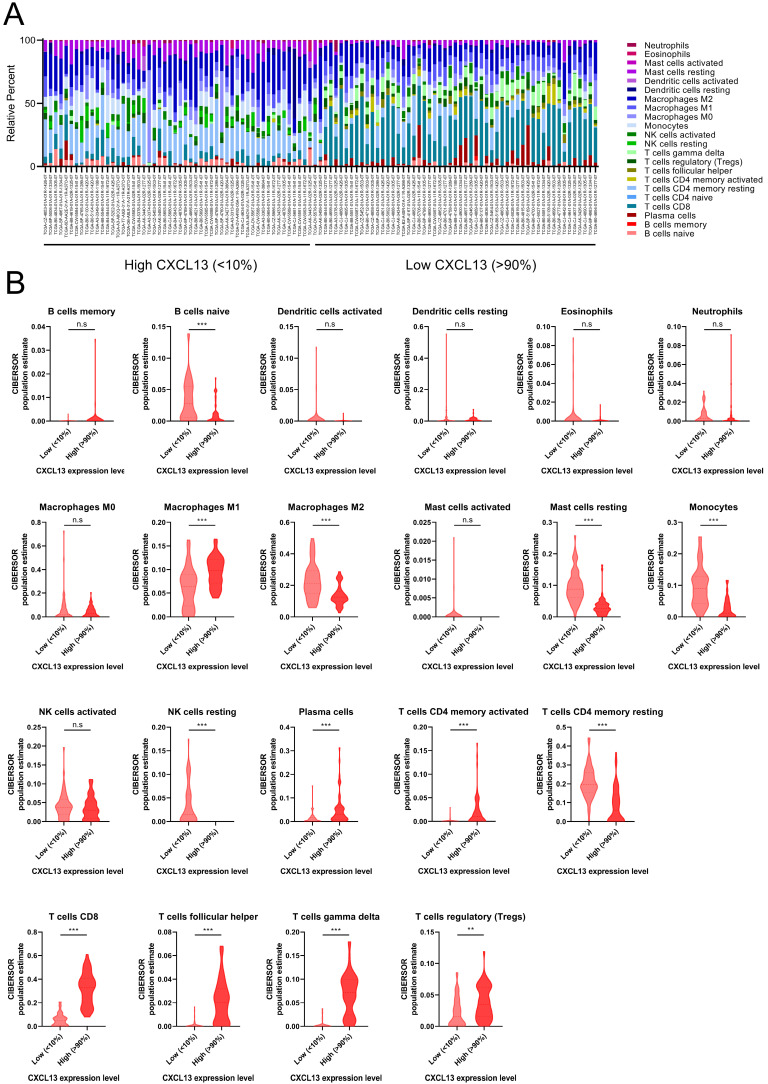
The landscape of immune infiltration in ccRCC (**A**) The difference of immune infiltration between top 10% highest-expressing and bottom 10% lowest-expressing CXCL13 samples. (**B**) The quantified contrast of the distribution of TIIC subtypes between top 10% highest-expressing and bottom 10% lowest-expressing CXCL13 samples *p < 0.05; **p < 0.01, ***p<0.001.

ccRCC patients from TCGA was divided into low (bottom 10% lowest-expressing CXCL13 samples) and high CXCL13 (top 10% highest-expressing CXCL13 samples) expression group. The differences between 22 subpopulations of TIICs in two groups were investigated using the CIBERSORT algorithm. We evaluated the average proportion of each immune cell type. Figure 5A was shown the details of the distribution of TIICs in both groups. There were large differences in the composition of TIICs in two groups. The results revealed that macrophages M1 cells were highly present in high CXCL13 ccRCC tissues, as well as plasma cells, CD4^+^ memory activated T cells, CD8^+^ T cells, follicular helper T cells, gamma delta T cells and Tregs (p<0.05). Moreover, the proportion of naïve B cells, macrophages M2 cells, resting mast cells, monocytes, resting NK cells and CD4^+^ memory resting T cells was higher in low CXCL13 expression tissues compared to high CXCL13 expression tissues (p<0.05).

**Figure 6:**
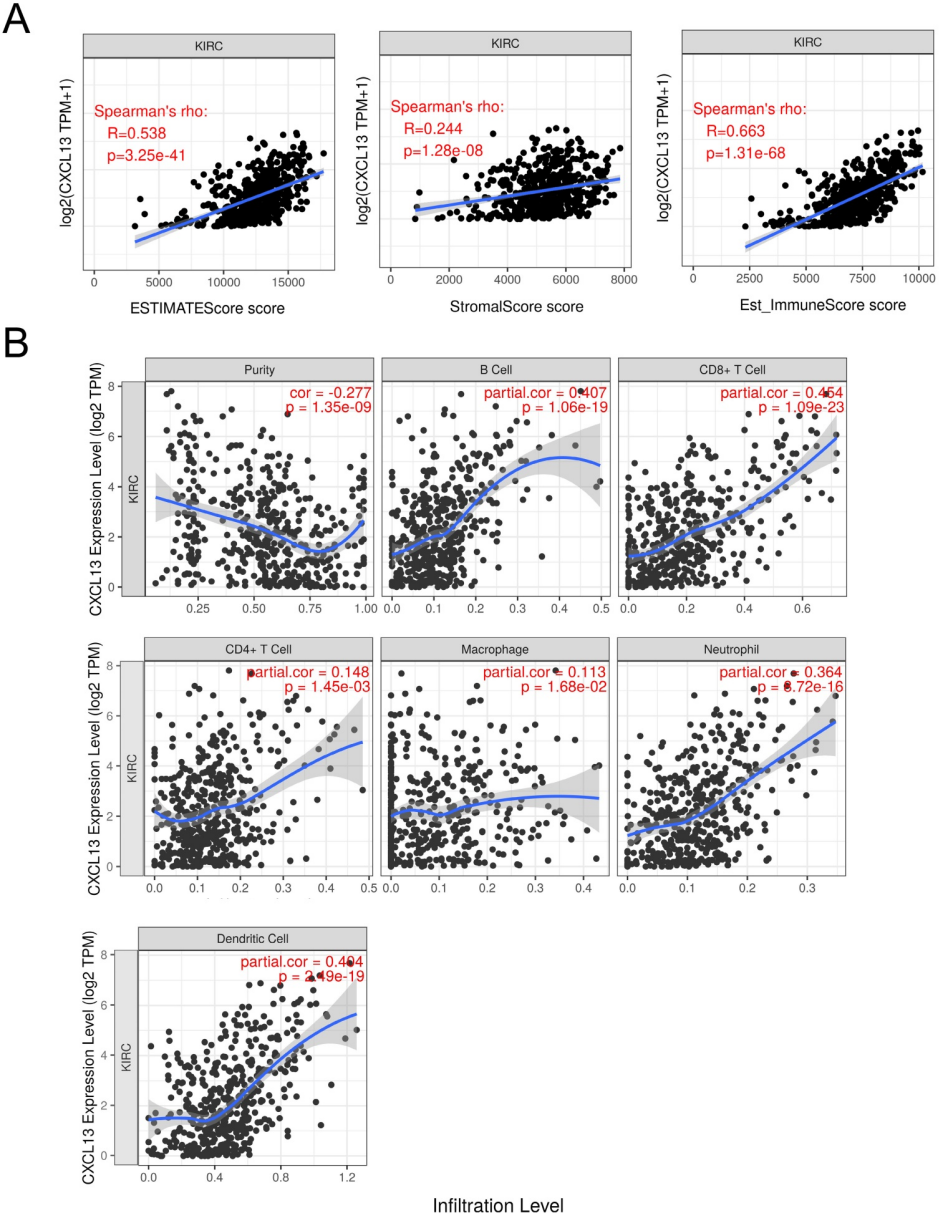
Correlation of CXCL13 expression with immune infiltration level in ccRCC. (**A**) The correlation of CXCL13 with ESTIMATE score, stromal score and immune score. (**B**) CXCL13 expression is significantly negatively related to tumor purity and has significant positive correlations with infiltrating levels of B cells, CD8^+^ T cells, CD4^+^ T cells, macrophages, neutrophils, and dendritic cells.

To further determine the correlation between CXCL13 and TIICs, ESTIMATE was used to evaluate immune score. As shown in Figure 6A, strong correlation was found in ESTIMATE score, stromal score and immune score (r=0.538, 0.244 and 0.663, respectively). TIMER analysis was used to clarify the correlation of CXCL13 expression with immune infiltration level in ccRCC, and the results revealed that CXCL13 expression was significantly negatively related to tumor purity and had significant positive correlations with infiltrating levels of B cells, CD8^+^ T cells, CD4^+^ T cells, macrophages, neutrophils, and dendritic cells (Figure 6B). Additionally, we analyzed the correlation between CXCL13 expression and immune marker genes of immune cells (Table 2), and we found that the expression levels of most marker sets of immune cells are correlated with CXCL13, except for M1 macrophage, natural killer cell, Th2 and Th17 cells.

### Identification of CXCL13 associated biological pathways

**Figure 7:**
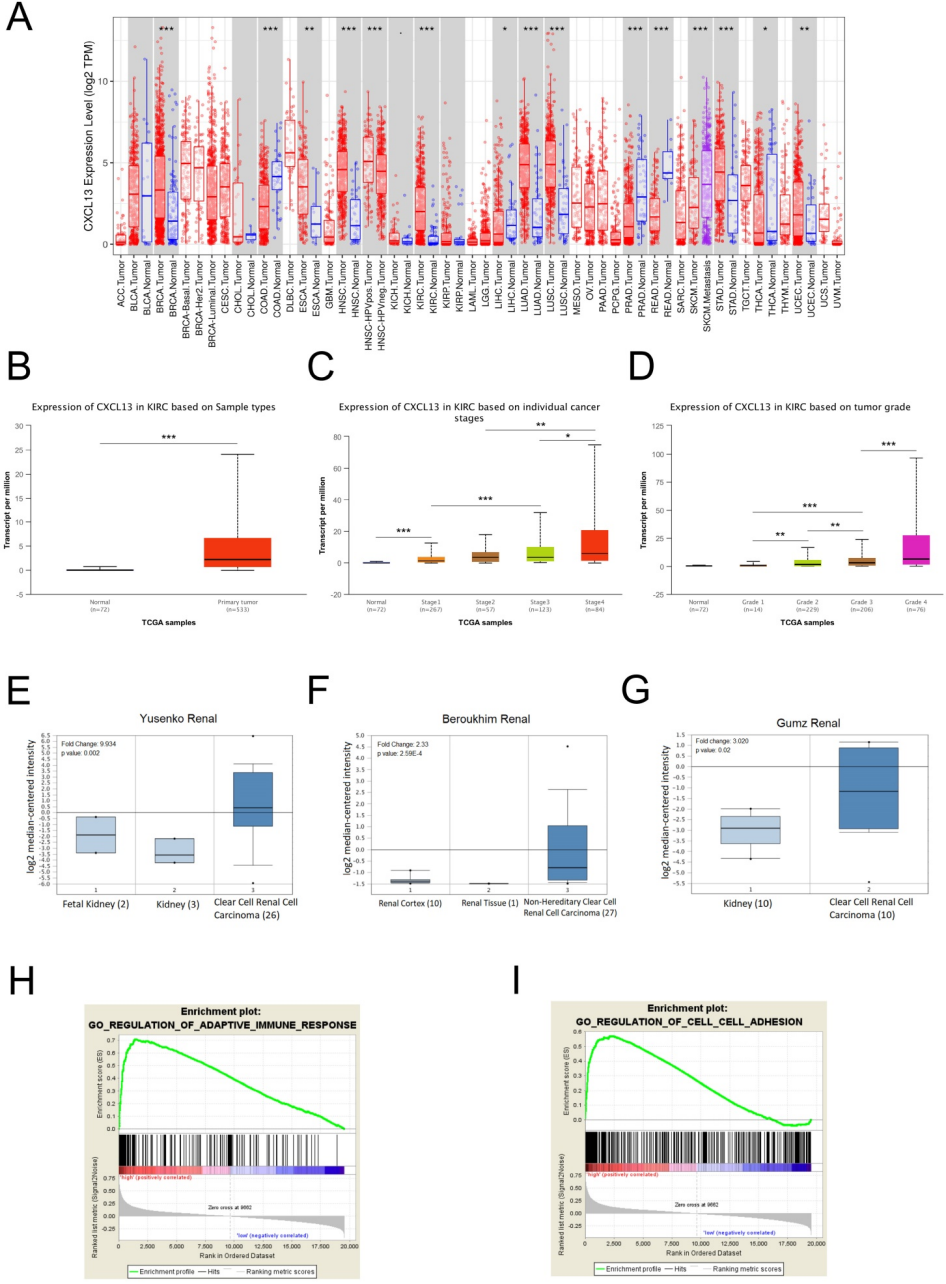
CXCL13 expression levels in ccRCC (**A**) Human CXCL13 expression levels in different types of tumor from TCGA database were determined by TIMER analysis. (**B**) CXCL13 expression level between non-tumor tissues and tumor tissues from ccRCC. (**C**) CXCL13 expression level among different tumor stages. (**D**) CXCL13 expression level among different tumor grades. (**E-G**) CXCL13 mRNA expression level in Oncomine database. (**H-I**) GSEA plot showed CXCL13 expression associated with immune response and cell adhesion processes *p < 0.05; **p < 0.01, ***p<0.001.

To further evaluate CXCL13 expression in various cancers, the differential CXCL13 expression between the tumor and normal tissues across all TCGA cohorts was shown in Figure 7A. Furthermore, CXCL13 expression was significantly higher in ccRCC (Figure 7B). Furthermore, CXCL13 was positively associated with tumor stage and tumor grade (Figure 7C-D). Additionally, CXCL13 mRNA levels in ccRCCs were analyzed using the Oncomine database. The results showed CXCL13 expression was higher in tumor tissues, compared to the normal tissues (Figure 7E-G).

According to the different CXCL13 expression levels, GSEA analysis, a robust computational method that determines whether the defined set of genes shows statistically significant differences, was used to determine biologic characteristics. Immune response and cell adhesion were enriched significantly, indicating that CXCL13 might play a vital role in these biological processes in ccRCC (Figure 7H-I).

### CXCL13 promotes migration ability in renal cell lines

**Figure 8:**
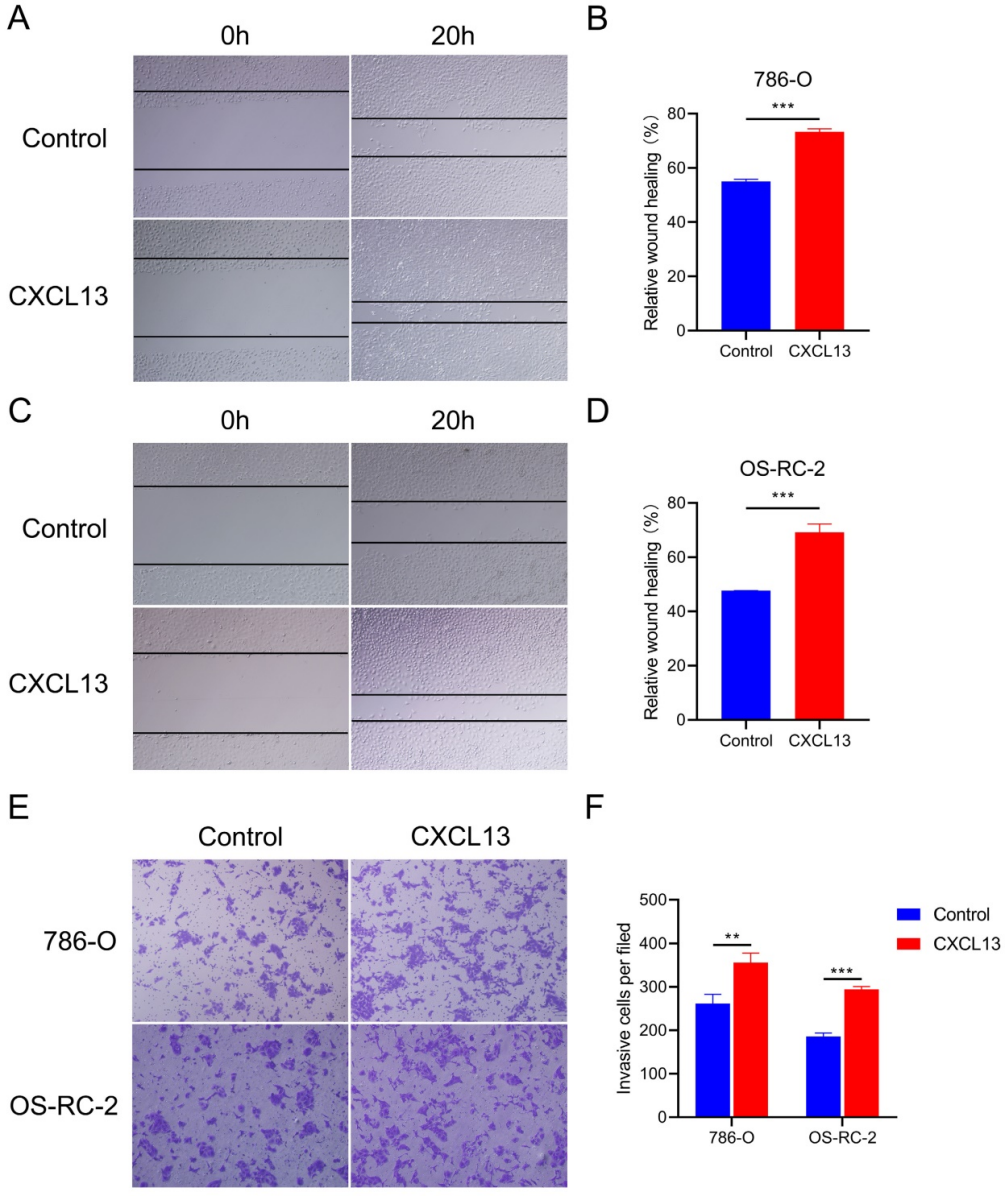
CXCL13 promote the migration ability of RCC. (**A-D**) The wound healing assays were used to examine the relative migration ability of CXCL13. Wound closure was observed after 20h. The normalized wound area of control cells at 0 h was set as 1. (**E-F**) Migration of 786-O and OS-RC-2 cells were measured by the transwell migration assay. Transwell assay was observed after 24h **p < 0.01, ***p<0.001.

To identify the effects of CXCL13 on the motility of RCC, wound healing assays were performed in 786-O and OS-RC-2 cells. The results showed that treatment with CXCL13 (50 ng/ml) significant increased the speed of wound closure in both cell lines (Figure 8A-D). Transwell assay showed a great increased migrating cells in CXCL13 treated cells (50 ng/ml), compared to control cells (Figure 8E-F). Taken together, these results indicated that CXCL13 significantly promoted the migration ability of RCC.

## Discussion

TCGA is an open access database which uses a genome-wide approach to reveal the genetic characteristics of cancers. Many studies of cancers such as RCC have screened diagnostic and prognostic biomarkers using TCGA 31, 32. Previous studies have shown that intrinsic tumor genes can cause changes in the tumor microenvironment 33, 34, which affect the occurrence and development of tumors, tumor progression, drug resistance, and overall prognosis 35-37. The RCC tumor microenvironment is unlike that of other tumor types 38; in most cancers, increased CD8^+^ T cell density is associated with improved prognosis 6, yet in RCC increased CD8^+^ T cell density is often associated with a worse outcome 39. A previous study which performed clustering analyses on the infiltrating immune cells in RCC, revealed a total of 17 macrophage and 22 T cell subsets 40. Therefore, the tumor immune microenvironment appears to play an important role in the occurrence and development of RCC.

In this study, we calculated immune cell and stromal cell scores using the ESTIMATE algorithm, finding that patients with a high immune cell score were likely to have a poor prognosis. Next, we identified 521 co-DEGs by comparing the gene expression of patients with high immune cell and stromal cell scores against those with low immune cell and stromal cell scores. GO term analysis revealed that the co-DEGs functions mainly involved the tumor microenvironment, such as T cell activation, immune response-regulating cell surface receptor signaling pathway, positive regulation of cytokine production, and cytokine activity. KEGG pathway enrichment analysis showed that the DEGs were enriched in cytokine-cytokine receptor interactions and the chemokine signaling pathway. Functional enrichment analysis confirmed that these DEGs were closely related to the RCC microenvironment. Next, we performed PPI network analysis and found 10 microenvironment-associated hub genes, including GPR55, CCR4, C3, CCL21, CCR7, CCL19, and CXCL13, which have been reported to promote proliferation, angiogenesis, migration, and invasiveness by altering the tumor microenvironment 41-44, and identified CCR4, GNG8 and CXCL13 were associated with prognosis of ccRCC patients. Furthermore, we evaluated the correlation between TIICs and CXCL13 and CXCL13 mRNA expression in ccRCC. GESA analysis enriched immune response and cell adhesion pathway, indicating that CXCL13 had potential role in tumor migration.

Chemokines are a family of chemotactic cytokines or ligands, which were related to direct migration of immune cells and tumor cells 45, 46. CXCL13, and its receptor, CXCR5 (CXCL13/CXCR5 axis), serve as an important pathway of tumor proliferation and metastasis 47-49. Previous studies demonstrated that CXCL13 upregulation was regarded as a biomarker for poor prognosis in various cancers, including ccRCC 50-52. Since high immune cell and stromal cell scores are associated with poor prognosis of ccRCC patients, we identified tumor microenvironment-associated genes that correlated with the prognosis of patients with ccRCC. We identified CXCL13 was associated with TIICs and enhanced the effect of migration.

Cancer cells are usually recognized by immune system, including innate and adaptive immunity. And the immune surveillance is driven by a network of mediators, such as cytokines and chemokines, et al 53. On one hand, chemokines regulate tumor microenvironment and initiation of antitumor immune response. On the other hand, chemokines could recruit immune-suppressive regulators and immunoinhibitory mediators to make immune escape. This means CXCL13 could act as a tumor-specific biomarker.

Previous studies had demonstrated that CXCL13 was associated with epithelial-to-mesenchymal transition (EMT) and could activate CXCR5/ERK pathway in breast cancer 48, 49. EMT is an important way highly associated with cell adhesion. Dysregulation of cell adhesion was associated with cancer progression and metastasis through promoting the motility of tumor cells and migration into adjacent tissues or invade vascular to distinct organs. Consistent with our results, CXCL13 expression level was positively correlated with immune response and cell adhesion by GSEA analysis. Wound healing and transwell assays showed CXCL13 promoted the migration ability in RCC.

However, there were several limitations in this study. First, the results analysis from TCGA have not detected in GEO database. Second, the detailed functions and potential mechanisms of CXCL13 in ccRCC are needed to be validated *in vitro* and *in vivo* experiment, which would be conducted in our further studies.

## Conclusions

In summary, we produced a list of tumor microenvironment-related genes and identified CXCL13 expression level correlated with poor prognosis and increased TIICs of ccRCC. Therefore, CXCL13 likely plays an important role in immune cell infiltration, acts as a prognosis biomarker in patients with ccRCC and has potential role in tumor migration. However, our findings need to be validated in future studies.

## Figures and Tables

**Table 1:** Patient characteristics and pathologic features

		Immune scores		*P*-value	StromalScore		*P*-value
Variables	Total patients	low	high		low	high	
**Age**	60.56± 12.14	60.77± 12.15	60.35±12.14		62.38±11.96	58.75±12.06	
				0.931			0.003
<60	245	122	123		105	140	
≥60	285	143	142		160	125	
**Gender**				0.069			0.084
female	186	103	83		103	83	
male	344	162	182		162	182	
**Stage**				0.011			0.11
stage I	265	151	114		132	133	
stage II	56	25	31		37	20	
stage III	123	55	68		59	64	
stage IV	82	32	50		37	45	
unknown	3	1	2		0	3	

**Table 2:** The association with immune cell markers and CXCL13 expression level

Description	Gene marker	r	P
CD8+ T cell	CD8A	0.5756	****
	CD8B	0.5212	****
T cell (general)	CD3D	0.589	****
	CD3E	0.6135	****
	CD2	0.6021	****
B cell	CD19	0.6094	****
	CD79A	0.4251	****
Monocyte	CD86	0.3852	****
	CD115 (CSF1R)	0.3022	****
M1 Macrophage	INOS (NOS2)	-0.06597	ns
	COX2(PTGS2)	-0.0151	ns
M2 Macrophage	CD163	0.2232	****
	VSIG4	0.3423	****
	MS4A4A	0.3002	****
Neutrophils	CD66b (CEACAM8)	0.008424	ns
	CD11b (ITGAM)	0.04541	ns
Natural killer cell	KIR2DL1	-0.03222	ns
	KIR2DL3	0.05152	ns
	KIR2DL4	0.3342	ns
	KIR3DL1	-0.0444	ns
	KIR3DL2	0.0731	ns
	KIR3DL3	0.05221	ns
Dendritic cell	HLA-DPB1	0.2912	****
	HLA-DQB1	0.2099	****
	HLA-DRA	0.2839	****
	HLA-DPA1	0.2647	****
Th1	T-bet (TBX21)	0.4259	****
	STAT4	0.4852	****
	STAT1	0.4578	****
	IFN-γ (IFNG)	0.6612	****
Th2	GATA3	0.0744	ns
	STAT6	-0.05662	ns
	IL13	0.1663	***
Tfh	BCL6	0.05778	ns
	IL21	0.4444	****
Th17	STAT3	0.02595	ns
	IL17A	0.01578	ns
Treg	FOXP3	0.5057	****
	CCR8	0.383	****
	TGFβ (TGFB1)	0.2035	****
T cell exhaustion	PD-1 (PDCD1)	0.6266	****
	CTLA4	0.6144	****
	LAG3	0.6436	****
	GZMB	0.5386	****

## Abbreviations

RCC: renal cell carcinoma
ccRCC: clear cell renal cell carcinoma
TIICs: tumor-infiltrating immune cells
ECM: extracellular matrix
TCGA: The Cancer Genome Atlas
PD-1: programmed death-1
PD-L1: programmed death-ligand 1
ESTIMATE: Estimation of Stromal and Immune cells in Malignant Tumor tissues using Expression data
DEGs: differentially expressed genes
FDR: false discovery rate
GO: gene ontology
KEGG: Kyoto Encyclopedia of Genes and Genomes
PPI: protein-protein interaction
GSEA: Gene set enrichment analysis
MSigDB: The Molecular Signatures Database
EMT: epithelial-to-mesenchymal transition
